# Interim PET response of Pola-R-CHP predicts outcome in previously untreated CD5-positive diffuse large B-cell lymphoma: a multicenter retrospective study

**DOI:** 10.1080/07853890.2026.2697132

**Published:** 2026-07-22

**Authors:** Jingrui Sui, Lijie Yan, Zengyan Liu, Peiqi Zhao, Xia Zhao, Zhihe Liu, Xiaofeng Luo, Jingjing Ye, Lijie Xing, Yuezhu Zhang, Junqing Xu

**Affiliations:** a Hematology Department, Affiliated Yantai Yuhuangding Hospital of Qingdao University, Yantai, China; b Hematology Department, Binzhou Medical University Hospital, Binzhou, China; c Lymphoma Department, Tianjin Medical University Cancer Institute, Tianjin, China; d Lymphoma Department, Affiliated Hospital of Qingdao University, Qingdao, China; e Hematology Department, Fujian Medical University Union Hospital, Fujian, China; f Hematology Department, Qilu Hospital of Shandong University, Jinan, China; g Lymphoma Department, Shandong Cancer Hospital and Institute, Shandong First Medical University and Shandong Academy of Medical Sciences, Jinan, China

**Keywords:** CD5-positive DLBCL, early therapeutic adjustment, interim partial response, Pola-R-CHP, progression-free survival

## Abstract

**Background:**

CD5-positive diffuse large B-cell lymphoma (DLBCL) is an aggressive subtype with poor outcomes. Following the approval of Pola-R-CHP in China, this study aimed to evaluate its efficacy as initial therapy for this high-risk population.

**Methods:**

We conducted a multicenter, retrospective study of previously untreated CD5-positive DLBCL patients who received Pola-R-CHP as first-line therapy between April 2023 and February 2025. Treatment response was assessed by PET/CT after 3 cycles and at the end of treatment.

**Results:**

Among 32 enrolled patients (median age 63.5 years), 78.1% had stage III–IV disease and 59.4% had an International Prognostic Index score ≥3. The overall response rate (ORR) was 100% after 3 cycles, with a complete response rate (CRR) of 71.9%. At end of treatment, the ORR and CRR were 93.8% and 75%, respectively. After a median follow-up of 17.9 months, 2-year progression-free survival (PFS) and overall survival rates (OS) were 70% and 82.7%. After adjusting covariates, failure to achieve a complete response (CR) after 3 cycles was a predictor of inferior survival [PFS HR: 18.1, *p* = 0.02; OS HR: 12.3, *p* = 0.033]. Grade 3–4 adverse events occurred in 31.3% of patients, with neutropenia being most common.

**Conclusions:**

CD5-positive DLBCL patients exhibited rapid and promising responses to first-line Pola-R-CHP with an acceptable safety profile. Interim response assessment is a potential prognostic factor. In instances where CR is not attained by the interim PET/CT, it is advisable to consider treatment modification, as this may indicate a potential failure to achieve CR.

## Introduction

CD5-positive diffuse large B-cell lymphoma (DLBCL) represents a distinct clinicopathological subtype accounting for approximately 10% of DLBCL, not otherwise specified (NOS) cases [1]. Emerging evidence suggests that CD5-positive DLBCL exhibits more aggressive biological behavior and poorer clinical outcomes compared to its CD5-negative counterpart [2]. Patients with CD5-positive DLBCL typically present with adverse clinical features including advanced age, higher disease stage, frequent extranodal involvement, non-germinal center B-cell (non-GCB) phenotype, and elevated International Prognostic Index (IPI) and Central Nervous System International Prognostic Index scores [3–5].

In the R-CHOP (rituximab, cyclophosphamide, doxorubicin, vincristine, and prednisone) treatment era, CD5-positive DLBCL patients consistently demonstrate inferior progression-free survival (PFS) and overall survival (OS) rates [2,6,7]. Research indicates that patients with CD5-positive DLBCL undergoing standard R-CHOP therapy exhibit two-year event-free survival and OS rates of 18% and 45%, respectively, which are inferior to those of their CD5-negative counterparts [2]. Furthermore, this subgroup exhibits a concerning propensity for central nervous system relapse, which significantly contributes to its dismal prognosis [8–10]. These clinical challenges highlight the urgent need for more effective therapeutic strategies for this high-risk population.

The development of polatuzumab vedotin (Pola), an anti-CD79b antibody–drug conjugate, has introduced a promising therapeutic option for DLBCL [11]. The Pola-R-CHP regimen (polatuzumab vedotin, rituximab, cyclophosphamide, doxorubicin, and prednisone) has shown superior efficacy compared to standard R-CHOP in previously untreated high-risk DLBCL patients [12].

One retrospective study suggests that Pola-R-mini-CHP therapy is effective and may improve outcomes in patients aged ≥80 years with CD5-positive DLBCL, achieving complete remission in five of six patients with no recurrences [13]. However, the specific clinical benefits of Pola-R-CHP in CD5-positive DLBCL, particularly regarding PFS and OS outcomes, remain to be fully elucidated.

This study aims to evaluate the efficacy and safety of Pola-R-CHP in CD5-positive DLBCL patients, with particular focus on the impact of interim treatment response on patient prognosis. The findings may provide valuable insights for optimizing treatment strategies for this challenging disease subtype [14].

## Methods

### Study design and patient selection

This retrospective, observational cohort study focused on patients with newly diagnosed CD5-positive DLBCL who received Pola-R-CHP as first-line therapy between April 2023 and February 2025. The research was conducted across six institutions: the Lymphoma Department of Tianjin Medical University Cancer Institute, the Hematology Department of Concord Hospital of Fujian Medical University, the Lymphoma Department of the Affiliated Hospital of Qingdao University, the Hematology Department of Qilu Hospital of Shandong University, the Lymphoma Department of Shandong Cancer Hospital and Institute, and the Hematology Department of the Affiliated Yantai Yuhuangding Hospital of Qingdao University. Eligible patients were those aged 18 years or older with newly diagnosed, histologically confirmed DLBCL exhibiting CD5 expression (≥30% of tumor cells) as determined by flow cytometry or immunohistochemistry, who underwent full-course first-line treatment with Pola-R-CHP during the specified timeframe. Exclusion criteria included primary central nervous system lymphoma, concurrent active malignancy, prior indolent lymphoma, or incomplete response assessment data. Our study adhered to the Declaration of Helsinki. The study received approval from the Institutional Review Boards of the Affiliated Yantai Yuhuangding Hospital of Qingdao University (No. 2025-917), and a waiver for informed consent was granted due to its retrospective nature.

#### Treatment regimen

Patients were administered rituximab at a dose of 375 mg/m^2^ intravenously on day 0, followed by cyclophosphamide at 750 mg/m^2^ intravenously on day 1, doxorubicin at 50 mg/m^2^ intravenously on day 1, and prednisone at 100 mg orally from days 1 to 5, with or without polatuzumab vedotin at a dose of 1.8 mg/kg intravenously on day 0.

### Data collection and outcomes

Comprehensive clinical data were systematically extracted from institutional electronic medical records, including baseline demographic characteristics, disease staging parameters (Ann Arbor staging system, IPI scores), clinical manifestations of B symptoms, diagnostic biomarkers (serum lactate dehydrogenase [LDH] levels, β2-microglobulin profiles), radiological evaluations *via* 18 F-FDG PET/CT, and histopathological confirmation through bone marrow biopsy. As per institutional practice, all patients underwent PET/CT imaging after 3 cycles of Pola-R-CHP (interim) and at the end of treatment. The scans were interpreted according to the Lugano 2014 criteria. This analysis is a post-hoc evaluation of these prospectively collected imaging data. Treatment-related adverse events (AEs) were graded according to CTCAE v5.0.

Outcomes included complete response rate (CRR), partial response (PR), overall response rate (ORR, CR + PR) defined by Lugano 2014 criteria. PFS was defined as time from treatment initiation to disease progression or death. OS was measured from diagnosis to death from any cause.

### Statistical analysis

Categorical variables were summarized as frequencies (%), continuous variables as medians and interquartile ranges. Categorical variables were compared using Chi-square or Fisher’s exact test. Survival curves (PFS, OS) were constructed using the Kaplan–Meier method and compared *via* log-rank test. Cox regression analyses were performed to investigate factors associated with outcomes, incorporating interim PET-CT result and key prognostic factors. Given the limited size of the cohort, this study was exploratory in nature and aimed to generate hypotheses. Statistical analysis was performed using SPSS 26, Prism 10 and R. A *p* value < 0.05 was considered significant. Swimlane plots were generated using ggplot2 in R.

## Results

### Patients characteristics

From April 2023 to February 2025, a total of 3,478 patients were initially screened. Among these, 2,341 were classified as other subtypes, 1,029 were CD5 negative, 65 received alternative frontline regimens, and 11 did not complete the full course of therapy, leading to their exclusion. Ultimately, 32 patients from six institutions were enrolled in our analysis (Supplementary Figure 1). Overall, the included and excluded groups were largely comparable, except that included patients had a higher proportion of patients with B symptoms (data are provided in Supplementary Table 1). The demographic and baseline characteristics of patients are summarized in Table 1.

**Table 1.** Demographic and baseline characteristics of CD5-positive diffuse Large B-cell lymphoma patients receiving Pola-R-CHP treatment.

	*N* = 32
Age, year, median and IQR ≤ 60 years > 60 years	63.5 (52–69.8) 14 (43.8) 18 (56.2)
Sex, *n* (%) Male Female	16 (50) 16 (50)
Ann Arbor, *n* (%) I–II III–IV	7 (21.9) 25 (78.1)
IPI, *n* (%) < 3 ≥ 3	13 (40.6) 19 (59.4)
B symptoms Yes No	22 (68.8) 10 (31.2)
Extranodal involvement, *n* (%) Yes No	22 (68.8) 10 (31.2)
Bone marrow involvement, n (%) Yes No	6 (18.8) 26 (81.2)
Bulky disease, *n* (%) Yes No	4 (12.5) 28 (87.5)
Elevated LDH, *n* (%) Yes No	23 (71.9) 9 (28.1)
Elevated β2 macroglobulin, *n* (%) Yes No	15 (46.9) 17 (53.1)
Cell of origin, *n* (%) GCB Non-GCB	8 (25) 24 (75)
Ki67, *n* (%) < 80% ≥ 80%	13 (40.6) 19 (59.4)
Double expression, *n* (%) Yes No	10 (31.3) 22 (68.7)
Double hit, *n* (%) Yes No Missing	2 (6.3) 13 (40.6) 17 (53.1)

Abbreviations: IQR, interquartile range; Pola-R-CHP, polatuzumab vedotin, rituximab, cyclophosphamide, doxorubicin, and prednisone; IPI, international prognostic index; LDH, lactate dehydrogenase; GCB, germinal center B‐cell subtype; non-GCB, non‐germinal center B‐cell subtype.

The median age was 63.5 years (interquartile range: 52–69.8), with an equal distribution of 50% females and 50% males. Approximately seventy-eight percent of patients presented with Ann Arbor stage III or IV disease. The majority (59.4%) exhibited an IPI score of 3 or higher, and 68.8% had extranodal involvements, predominantly affecting the spleen. Tumor burden indicators, such as elevated LDH and β2-microglobulin levels, were present in 71.9% and 46.9% of patients, respectively. Additionally, 75% of patients were classified as non-germinal center B-cell subtype according to the Hans algorithm [15], and 31.3% were diagnosed with double-expressor lymphoma. Fifteen patients were tesed for C-myc and BCL-2 FISH assay, and two patients were diagnosed with double hit lymphoma.

### Efficacy

**Figure 1.**
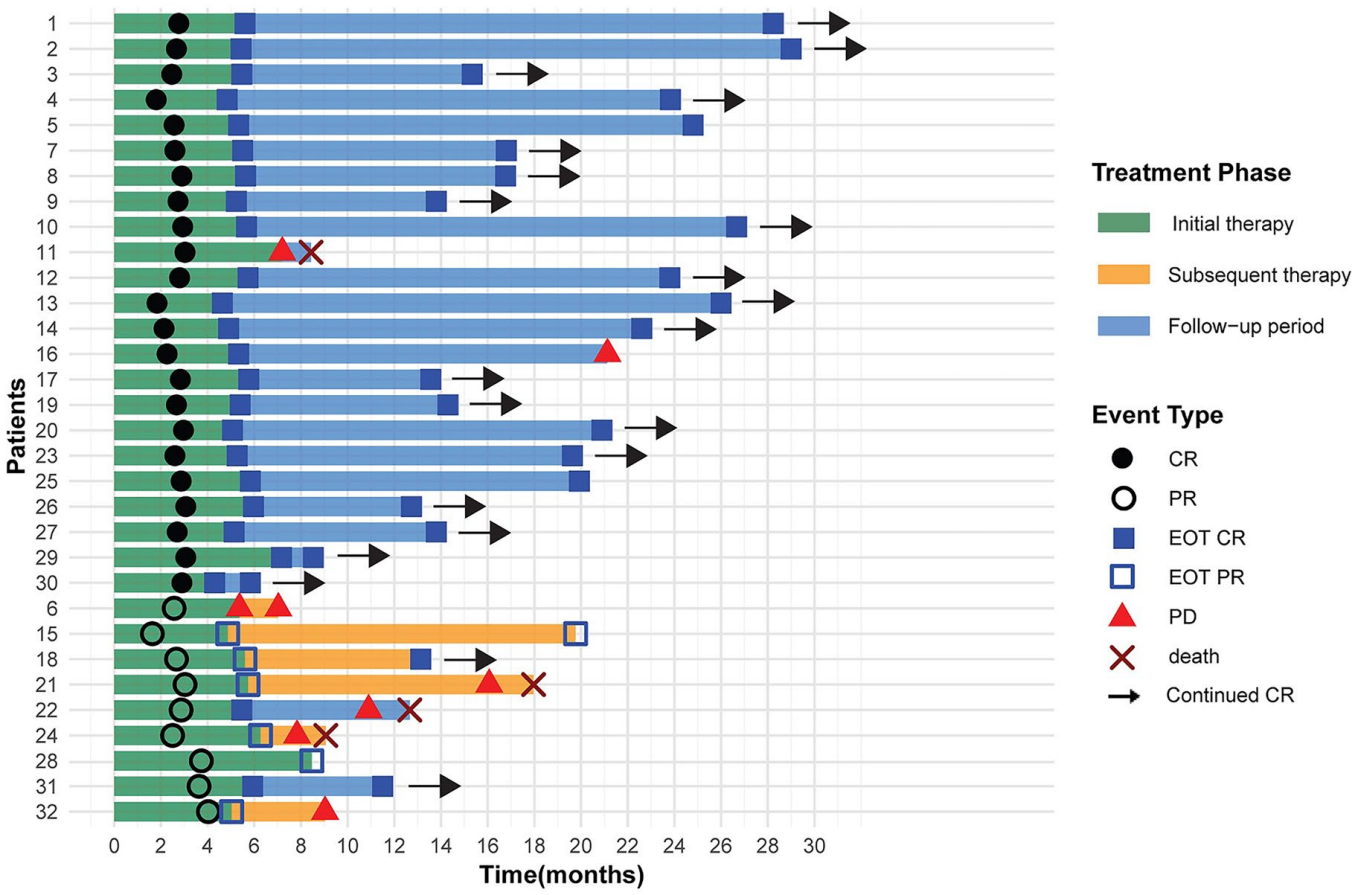
Swimmer plot of patients enrolled in the study. Each row represents a patient and the length of each bar represents the time from treatment initiation to the most recent follow-up time. CR, complete response; PR, partial response; PD, progressive disease; EOT CR, end of treatment complete response; EOT PR, end of treatment partial response; Continued CR, continued complete response.

The efficacy of CD5-positive DLBCL patients to Pola-R-CHP is summarized in Table 2. Interim efficacy assessed by PET-CT showed that 23 patients (71.9%) achieved complete response (CR) and 9 patients (28.1%) achieved PR, resulting in an ORR of 100% and a CRR of 71.9%. Subsequently, after completing treatment cycles, efficacy evaluation revealed 24 patients (75%) with CR, 6 patients (18.8%) with PR, and 2 patients (6.2%) with progressive disease (PD). At this stage, the ORR and CRR were 93.8% and 75%, respectively. During the entire treatment course, the best overall response was observed with an ORR of 100% and a CRR of 78.1%. Among the 32 patients, 16 received lumbar puncture for intrathecal injection, and none developed central nervous system (CNS) involvement. Besides, no patient received consolidative ASCT after completion of Pola-R-CHP. Swimmer plot of patients enrolled in the study was shown in Figure 1.

**Table 2.** The efficacy of CD5-positive diffuse Large B-cell lymphoma to Pola-R-CHP at different treatment time points.

Efficacy	Time point		
	Interim assessment	End of treatment	Best overall response
CRR, *n* (%) [95% CI]	23 (71.9) [61.2–89]	24 (75) [57.9–86.7]	25 (78.1) [61.2–89]
PR, *n* (%) [95% CI]	9 (28.1) [15.8–45.4]	6 (18.8) [8.9–35.3]	7 (21.9) [11–38.8]
PD, *n* (%) [95% CI]	0 (0) [0–10.7]	2 (6.2) [1.1–20.1]	0 (0) [0–10.7]
ORR, *n* (%) [95% CI]	32 (100) [89.3–100]	30 (93.8) [79.9–98.9]	32(100) [89.3–100]

Abbreviations: Pola-R-CHP, polatuzumab vedotin, rituximab, cyclophosphamide, doxorubicin, and prednisone; CRR, complete remission rate; 95% CI, 95% confidence interval; PR, partial response; PD, progressive disease; ORR, overall response rate.

### Subsequent antilymphoma therapy

For patients who failed to attain CR at the end of treatment assessment or experienced relapse post initial treatment, salvage therapeutic interventions were administered. As of the data cutoff date, the administration rate of subsequent antilymphoma therapy was 6 patients (18.8%).

### PFS, OS and duration of complete response (DoCR)

**Figure 2.**
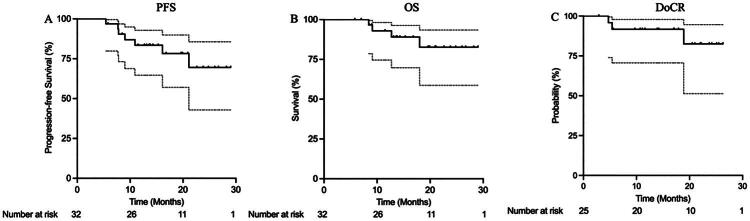
The PFS (A), OS (B) and DoCR (C) of CD5-positive diffuse large B-cell lymphoma treated with Pola-R-CHP. PFS, progression-free survival; OS, overall survival; DoCR, duration of complete response; Pola-R-CHP, polatuzumab vedotin, rituximab, cyclophosphamide, doxorubicin, and prednisone.

The PFS, OS and DoCR outcomes of CD5-positive DLBCL patients treated with Pola-R-CHP are summarized in Table 3 and Figure 2. Following a median follow-up period of 17.9 months, the PFS rate for these patients was 78.1% (95% CI, 61.2-89). The 1-year PFS rate was 83.5% (95% CI, 71.2–97.9), whereas the 2-year PFS rate remained stable at 70% (95% CI, 51.1–94.6). Regarding OS, the rate at the conclusion of follow-up was 87.5% (95% CI, 71.9–95). The 1-year OS rate was 92.9% (95% CI, 84–100), and the 2-year OS rate was 82.7% (95% CI, 68–100). Given the median follow-up of 17.9 months, the 2-year PFS and OS estimates remain immature and should be interpreted with caution. All patient deaths were attributed to disease progression.

**Table 3.** Progression-free survival, overall survival and duration of complete response of CD5-positive diffuse large B-cell lymphoma patients treated with Pola-R-CHP.

Outcome	
PFS (*N* = 32) Patients who had progression or relapse, *n* (%) 1-year (95% CI, %) 2-year (95% CI, %)	7 (21.9) 83.5 (71.2–97.9) 70 (51.1–94.6)
OS (*N* = 32) Patients who died, *n* (%) 1-year (95% CI, %) 2-year (95% CI, %)	4 (12.5) 92.9 (84–100) 82.7 (68–100)
DoCR (*N* = 25) Patients who had progression or relapse after CR, *n* (%) 1-year (95% CI, %) 2-year (95% CI, %)	3 (12) 91.7 (81.3–100) 82.5 (64.9–100)

Abbreviations: Pola-R-CHP, polatuzumab vedotin, rituximab, cyclophosphamide, doxorubicin, and prednisone; PFS, progression-free survival; OS, overall survival; DoCR, duration of complete response; CR, complete response; 95% CI, 95% confidence interval.

With a median follow-up of 16.8 months, among the 25 patients who had achieved CR at either the interim or end-of-treatment assessment, 22 patients (88%) maintained CR, while 3 patients (12%) experienced relapse. Additionally, the DoCR rate for these patients was 88% (95% CI, 70–95.8), and the 1-year and 2-year DoCR rates were 91.7% (95 CI, 81.3–100) and 82.5% (64.9–100), respectively. The median DoCR was not reached.

### End-of-treatment outcomes in patients with interim PR

**Figure 3.**
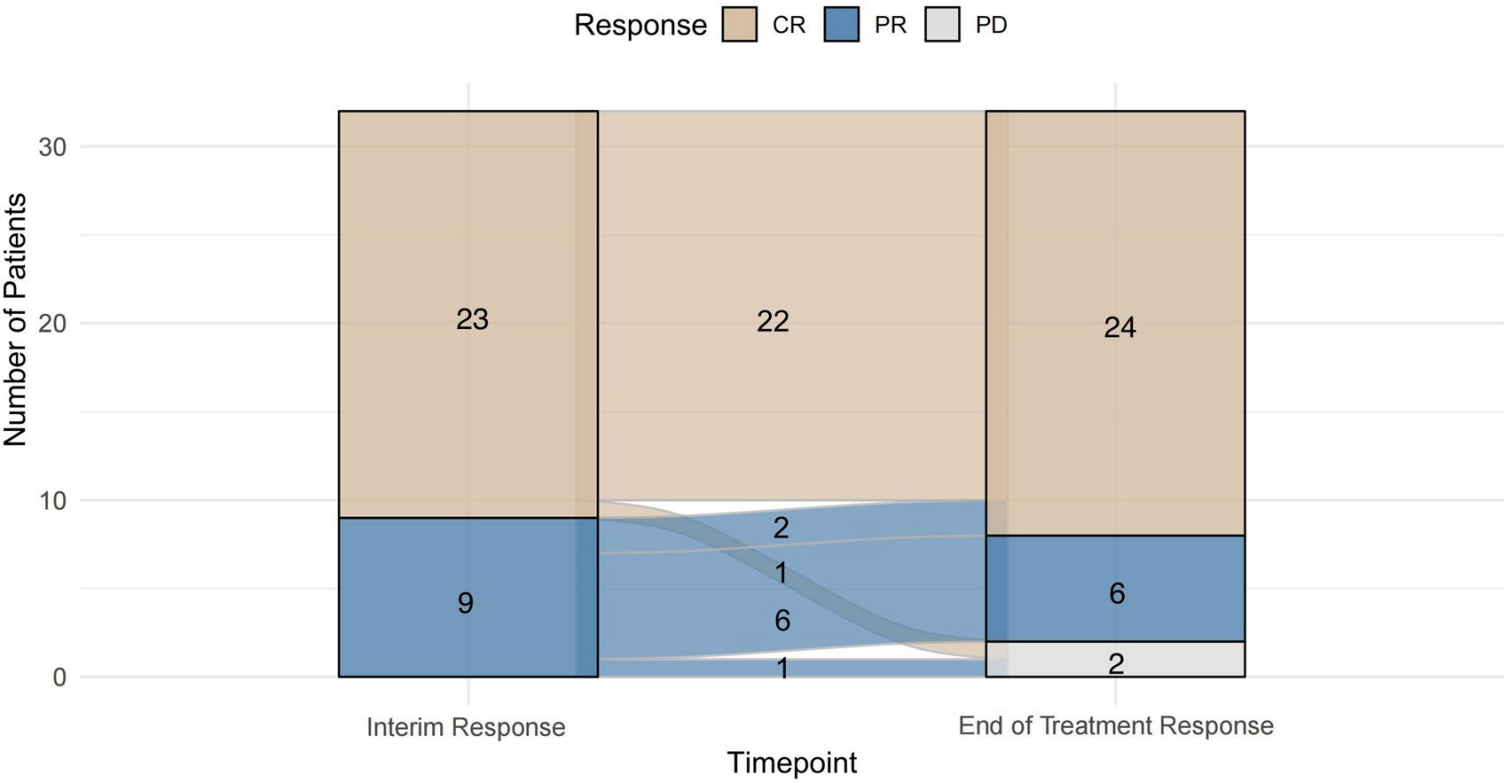
Response transition at interim and end of treatment in CD5-positive diffuse large B-cell lymphoma patients treated with Pola-R-CHP. CR, complete response; PR, partial response; PD, progressive disease; Pola-R-CHP, polatuzumab vedotin, rituximab, cyclophosphamide, doxorubicin, and prednisone.

Notably, patients who achieved interim PR cohort exhibited distinct response profiles at the end of treatment (Figure 3). Among the 9 patients who attained PR at interim assessment: 2 converted to CR by the end of treatment, 6 maintained PR, and 1 experienced PD.

Among patients who achieved an interim PR and later attained a CR by the end of treatment, both had stage IV disease, high-risk IPI scores of 4 to 5, B symptoms, bone marrow, extranodal involvement and elevated LDH levels. In contrast, among the seven patients who achieved either a PR (*n* = 6) or PD (*n* = 1) after six cycles, the median age was 69 years, with four females and three males. All these patients presented with stage III or IV disease (four with stage III and three with stage IV), high-risk IPI scores ranging from 2 to 4, and extranodal involvement. B symptoms were observed in four patients, bone marrow infiltration in two, and elevated LDH levels in two. Six patients in this group had the non-GCB subtype, and Ki-67 expression ranged from 60% to 80%.

However, among the 23 patients who achieved CR after 3 cycles, 22 maintained remission at the final PET/CT assessment, one relapsed from CR by end-of-treatment evaluation.

**Figure 4.**
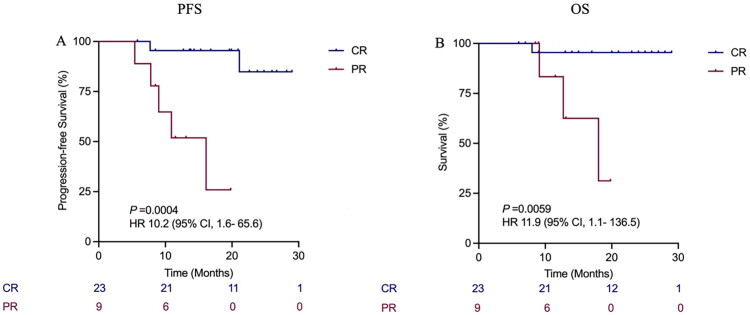
Differences in PFS (A) and OS (B) between patients who achieved CR compared to those with PR at interim assessment among those treated with Pola-R-CHP for CD5-positive diffuse large B-cell lymphoma. PFS, progression-free survival; OS, overall survival; CR, complete response; PR, partial response.

**Figure 5.**
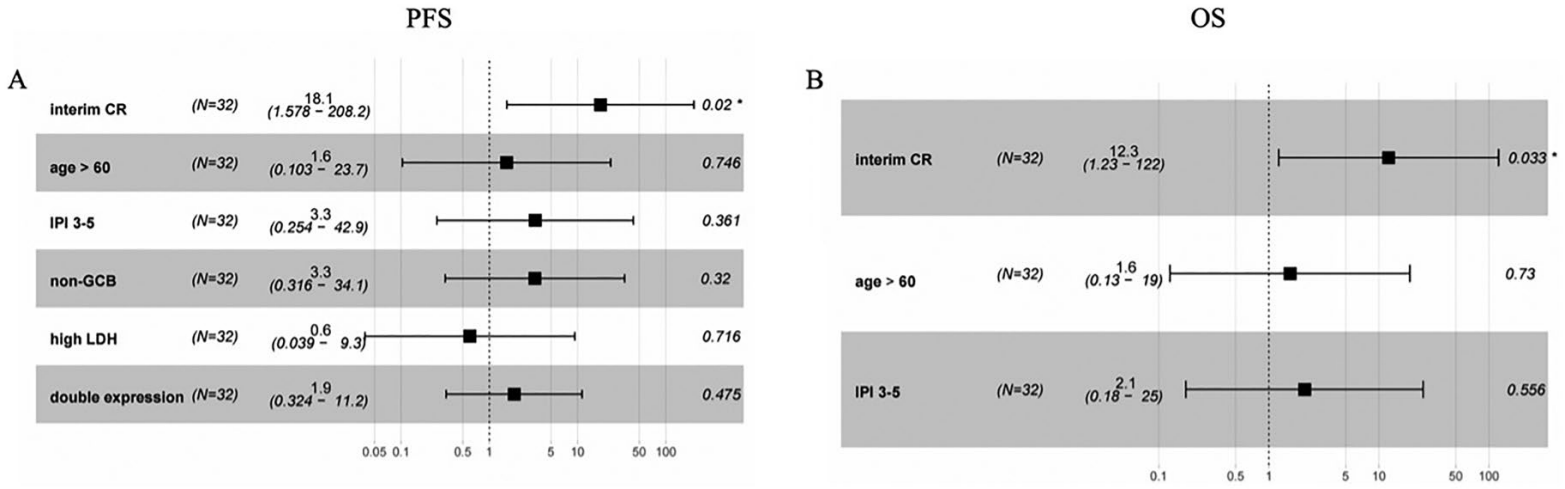
PFS (A) and OS (B) forest plots in CD5+ DLBCL patients treated with Pola-R-CHP. Univariate Cox regression analyses show interim CR predicts superior PFS and OS in CD5+ DLBCL patients treated with Pola-R-CHP. PFS, progression-free survival; OS, overall survival.

Moreover, patients with interim PR demonstrated worse outcome than those achieved interim CR. After adjusting covariates, failure to achieve a CR after 3 cycles was a potential prognostic factor of inferior PFS [hazard ratio (HR), 95% CI 18.1 (1.6–208.2), *p* = 0.02] and OS [HR, 95% CI 12.3 (1.2-122), *p* = 0.033]. Data are shown in Figures 4 and 5.

Based on the data above, Pola-R-CHP demonstrated enhanced risk stratification capability in CD5-positive DLBCL patients. It achieves this by identifying, during interim assessments, patients who achieved PR with a lower likelihood of reaching CR by end of treatment and inferior outcomes during follow-up, potentially indicating the need for early therapeutic adjustments.

### Safety profiles

Treatment was well tolerated. And 10 out of 32 patients (31.3%) experienced Grade 3 and 4 AEs with neutropenia (4, 12.5%) being the predominant issue. Additional reported ≥3 AEs comprised thrombocytopenia (2, 6.3%), anemia (3, 9.4%), febrile neutropenia (2, 6.3%). And other non-hematologic adverse reactions included dyspepsia (2, 6.3%) and peripheral neuropathy (2, 6.3%). There were no treatment related deaths. Additionally, prophylactic use of pegylated granulocyte colony-stimulating factor or granulocyte colony-stimulating factor in some patients during treatment further reduced the risk of infections associated with neutropenia. All the detailed AEs are summarized in Table 4.

**Table 4.** Adverse events in CD5-positive diffuse large B-cell lymphoma patients receiving Pola-R-CHP.

AEs	Patients with events, *n* (%)	
	All grades	Grades 3–4
Overall	20 (62.5)	10 (31.3)
Neutropenia	11 (34.4)	4 (12.5)
Thrombocytopenia	7 (21.9)	2 (6.3)
Anemia	10 (31.3)	3 (9.4)
Febrile neutropenia	2 (6.3)	2 (6.3)
Dyspepsia	8 (25)	2 (6.3)
Pneumonia	3 (9.4)	1 (3.1)
Peripheral sensory neuropathy	10 (31.3)	2 (6.3)

Abbreviations: Pola-R-CHP, polatuzumab vedotin, rituximab, cyclophosphamide, doxorubicin, and prednisone; AEs, adverse events.

## Discussion

This multicenter, retrospective study offers some of the initial real-world evidence supporting the efficacy and safety of Pola-R-CHP as a first-line treatment for CD5-positive DLBCL, a distinct subtype known for its aggressive clinical course, high rates of extranodal involvement, and poor outcomes with standard therapy [2,16,17]. The patient population in our cohort exhibited this high-risk profile, with a majority presenting with advanced Ann Arbor stage (III–IV), elevated IPI scores, and a significant prevalence of the non-GCB subtype according to the Hans algorithm – all characteristics consistently linked to inferior survival in CD5-positive DLBCL [3,5]. However, none of the patients were observed with CNS involvement in our cohort which is inconsistent with previously reported high rate of CNS involvement in CD5-positive DLBCL, a finding that may be partially attributed to CNS prophylaxis.

The central finding of our study is the remarkable efficacy of Pola-R-CHP in this challenging patient population. We observed an unprecedented interim ORR of 100% after 3 cycles, accompanied by a CRR of 71.9%. The best-observed CRR of 78.1% throughout the treatment course compares favorably with historical data for CD5-positive DLBCL treated with R-CHOP, where complete response rates are typically reported between 40% and 60%, and median overall survival is generally less than 2 years [17,18]. The superior efficacy of Pola-R-CHP over R-CHOP in the broader DLBCL population was established in the pivotal POLARIX trial [12], and our data suggest that this benefit may be particularly pronounced in the CD5-positive high-risk subgroup. The mechanism underlying this enhanced activity may involve the targeted delivery of monomethyl auristatin E (MMAE) by polatuzumab vedotin. By binding to CD79b, a component of the B-cell receptor, the antibody-drug conjugate is internalized and releases its cytotoxic payload, potentially bypassing or overcoming key resistance pathways, such as constitutive B-cell receptor signaling and survival pathways, which are often dysregulated in aggressive B-cell lymphomas [19,20].

The most critical and clinically actionable insight from our analysis is the potential prognostic value of the interim PET/CT assessment beyond traditional risk factors. We demonstrated that patients who did not achieve a CR after just 3 cycles exhibited markedly worse PFS and OS compared to those who did achieve a CR. This finding is consistent with the expanding literature on response-adapted therapy in DLBCL, which suggests that early response serves as a surrogate for chemosensitivity and long-term outcomes [21,22]. In the context of CD5-positive DLBCL, an interim PR likely identifies a patient subgroup with de facto chemorefractory disease, for whom continuation of the same therapy may prove futile [23]. Our data thus support a compelling case for a paradigm shift. We contend that an interim PR on Pola-R-CHP should not be regarded merely as a ‘slow response’, but rather as a potentially strong indication for an early and proactive modification of the treatment strategy. For these high-risk patients, options may include transitioning to salvage regimens that incorporate other novel agents, such as the BTK inhibitor ibrutinib, which has demonstrated activity in CD5-positive DLBCL [24,25], or T-cell engaging therapies like chimeric antigen receptor (CAR) T-cell therapy or bispecific antibodies [26,27], potentially serving as a bridge to consolidative autologous stem cell transplantation [28].

The safety profile of Pola-R-CHP in our real-world cohort was manageable and aligned with the established toxicity spectrum of the regimen observed in clinical trials [11,12]. Neutropenia emerged as the most prevalent high-grade adverse event, highlighting the necessity for diligent hematological monitoring and supportive care. Although peripheral neuropathy was observed, which is a recognized class effect of MMAE-based antibody-drug conjugates [29], it remained generally manageable.

Our study has several limitations. First, although the sample size is substantial for this rare entity, it remains modest. Second, the multivariable analyses rely on a limited number of events, resulting in extremely wide confidence intervals; therefore, these analyses are exploratory in nature. Third, while our cohort reflects real-world practice, the exclusion of patients receiving alternative regimens may limit the generalizability of our findings to broader populations. Additionally, the median follow-up time was 17.9 months, which is relatively short. Extended follow-up is necessary to confirm the durability of treatment responses and to provide a more accurate estimation of long-term survival outcomes.

In conclusion, our findings position Pola-R-CHP as a highly promising first-line therapy for CD5-positive DLBCL, demonstrating the ability to induce deep and rapid remissions. More importantly, we establish interim PET/CT after 3 cycles as a potential decision point. Failure to achieve an interim CR identifies a patient subgroup with a poor prognosis, treatment modifications may be justified. Future prospective, randomized studies are necessary to formally validate this response-adapted approach and to define the optimal alternative regimens for this ultra-high-risk patient population [17,30].

## Conclusions

CD5-positive DLBCL patients exhibited rapid and promising responses to first-line Pola-R-CHP with an acceptable safety profile. Interim response assessment is a potentially critical prognostic indicator. In cases where CR is not attained by the interim PET/CT, it is advisable to consider treatment modification, as this may indicate a potential failure to achieve CR. Due to the elevated risk of disease relapse in these patients, altering the therapeutic approach may also be justified.

## Supplementary Material

Supplementary Figure 1.jpg

## Data Availability

The data that support the findings of this study are available from the corresponding author upon reasonable request.
