# Supplementary figures and images for: Interim PET response of Pola-R-CHP predicts outcome in previously untreated CD5-positive diffuse large B-cell lymphoma: a multicenter retrospective study
Source: Ann Med. 2026 Jul 22;58(1):2697132. doi: 10.1080/07853890.2026.2697132 (PMC13393052; doi:10.1080/07853890.2026.2697132)

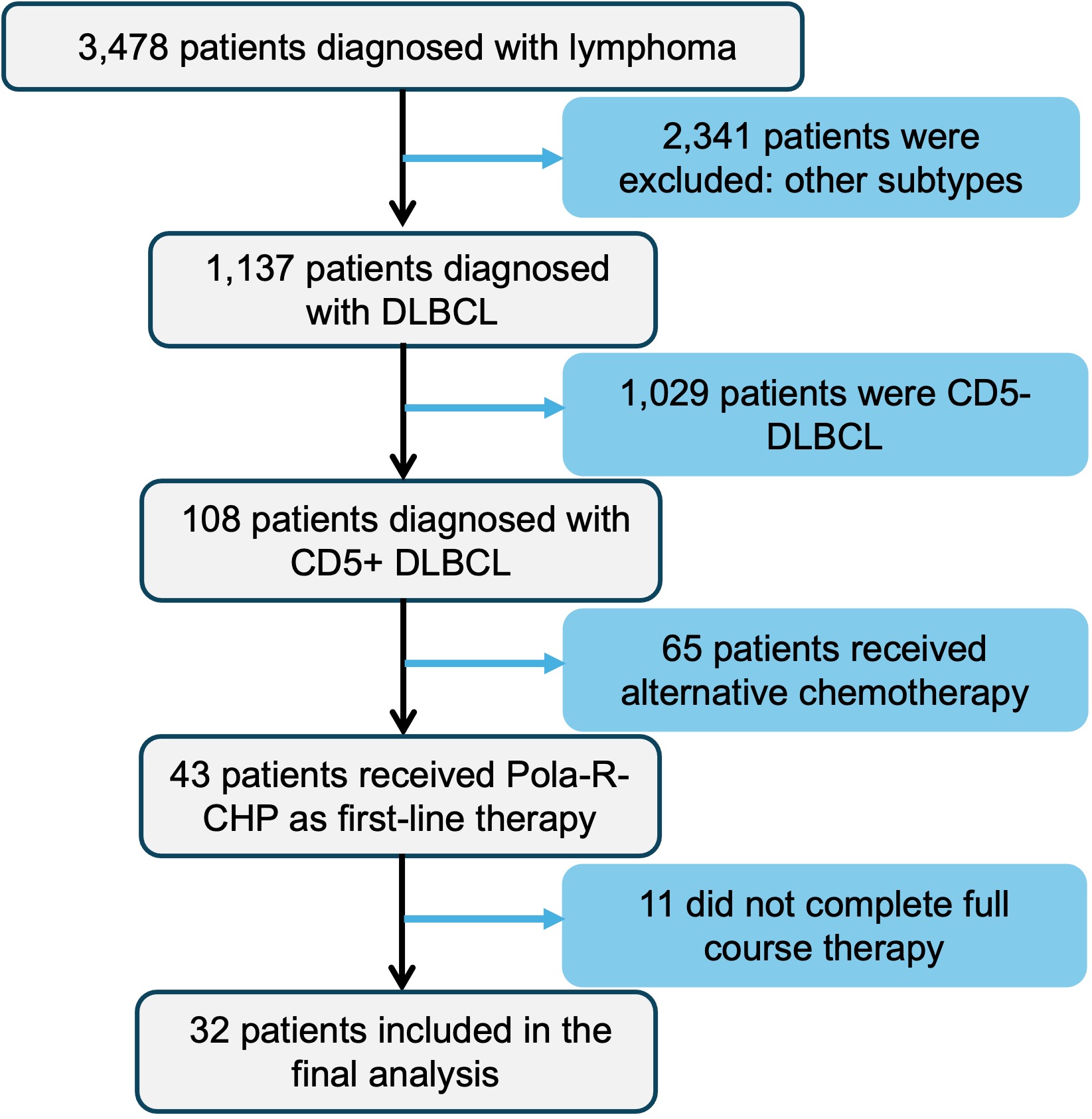

Supplement: Supplementary Figure 1.jpg [file IANN_A_2697132_SM9552.jpg]
